# Long-Term Impact of the World Bank Loan Project for Schistosomiasis Control: A Comparison of the Spatial Distribution of Schistosomiasis Risk in China

**DOI:** 10.1371/journal.pntd.0001620

**Published:** 2012-04-17

**Authors:** Zhijie Zhang, Rong Zhu, Michael P. Ward, Wanghong Xu, Lijuan Zhang, Jiagang Guo, Fei Zhao, Qingwu Jiang

**Affiliations:** 1 Department of Epidemiology and Biostatistics, School of Public Health, Fudan University, Shanghai, People's Republic of China; 2 Key Laboratory of Public Health Safety, Ministry of Education, Shanghai, People's Republic of China; 3 Laboratory for Spatial Analysis and Modeling, School of Public Health, Fudan University, Shanghai, People's Republic of China; 4 National Institute of Parasitic Diseases, Chinese Center for Disease Control and Prevention, Shanghai, People's Republic of China; 5 Faculty of Veterinary Science, The University of Sydney, Sydney, New South Wales, Australia; Centre Suisse de Recherches Scientifiques, Cote d'Ivoire

## Abstract

**Background:**

The World Bank Loan Project (WBLP) for controlling schistosomiasis in China was implemented during 1992–2001. Its short-term impact has been assessed from non-spatial perspective, but its long-term impact remains unclear and a spatial evaluation has not previously been conducted. Here we compared the spatial distribution of schistosomiasis risk using national datasets in the lake and marshland regions from 1999–2001 and 2007–2008 to evaluate the long-term impact of WBLP strategy on China's schistosomiasis burden.

**Methodology/Principal Findings:**

A hierarchical Poisson regression model was developed in a Bayesian framework with spatially correlated and uncorrelated heterogeneities at the county-level, modeled using a conditional autoregressive prior structure and a spatially unstructured Gaussian distribution, respectively. There were two important findings from this study. The WBLP strategy was found to have a good short-term impact on schistosomiasis control, but its long-term impact was not ideal. It has successfully reduced the morbidity of schistosomiasis to a low level, but can not contribute further to China's schistosomiasis control because of the current low endemic level. A second finding is that the WBLP strategy could not effectively compress the spatial distribution of schistosomiasis risk. To achieve further reductions in schistosomiasis-affected areas, and for sustainable control, focusing on the intermediate host snail should become the next step to interrupt schistosomiasis transmission within the two most affected regions surrounding the Dongting and Poyang Lakes. Furthermore, in the lower reaches of the Yangtze River, the WBLP's morbidity control strategy may need to continue for some time until snails in the upriver provinces have been well controlled.

**Conclusion:**

It is difficult to further reduce morbidity due to schistosomiasis using a chemotherapy-based control strategy in the lake and marshland regions of China because of the current low endemic levels of infection. The future control strategy for schistosomiasis should instead focus on a snail-based integrated control strategy to maintain the program achievements and sustainably reduce the burden of schistosomiasis in China.

## Author Summary

Schistosomiasis japonica is an important disease in China with a documented history of more than 2,100 years. The World Bank Loan Project (WBLP) implemented during 1992–2001 contributed greatly to China's schistosomiasis control. This study shows that the long-term impact of WBLP strategy on schistosomiasis control was not ideal. It can only maintain the morbidity of schistosomiasis at a low level, but can not reduce it further. Also, the WBLP strategy could not effectively compress the spatial distribution of schistosomiasis risk. To achieve further reductions in schistosomiasis-affected areas, and for sustainable control, focusing on controlling the intermediate host snail in the lake and marshland regions was suggested to be the next step to interrupt schistosomiasis transmission within the two most affected regions surrounding the Dongting and Poyang Lakes. While in the lower reaches of the Yangtze River, the WBLP's morbidity control strategy may need to continue for some time until snails in the upriver provinces have been well controlled.

## Introduction

Schistosomiasis japonica, a disease caused by the trematode *Schistosoma japonicum*, has a documented history of more than 2100 years in China [1]. It severely impacts the health of residents within endemic areas, causing substantial morbidity such as wasting, weakness, ascites and growth retardation [1], [2], [3]. Recognizing the large public health and socio-economic impact of this disease, the government of China initiated a large-scale schistosomiasis control program in the mid-1950s and achievements have been monitored during nearly 60 years of continuous endeavor [2], [4], [5]. At present, the schistosomiasis endemic regions have been largely reduced and confined to seven provinces along the Yangtze River: five provinces of Hunan, Hubei, Anhui, Jiangxi and Jiangsu in the lake and marshland regions and two in the mountainous regions, Yunnan and Sichuan provinces [6], [7], [8]. Many projects or programs have contributed to this success. Among others, the World Bank Loan Project (WBLP) targeted at schistosomiasis control in China has played an important role during the period of 1992–2001. Zhang&Wong [9] and Chen et al. [10] evaluated the impact of the WBLP strategy before and shortly after the end of the project. These authors concluded that the original objectives of the WBLP strategy– to control schistosomiasis morbidity – had been met, but that snail infested areas had increased to a certain degree and that snail infection fluctuated at low levels. After the termination of the WBLP strategy, there was a consistent gap between available funding and the financial resources required to maintain program achievements and to make further progress [10]. Many researchers have reported that schistosomiasis prevalence has rebounded in some regions, even where the criteria of transmission interruption or control had been previously met [11], [12]. A national survey carried out in 2004 confirmed the re-emergence of schistosomiasis in China [13]. This led us to question the long-term impact of the WBLP strategy and investigate a more sustainable strategy for schistosomiasis control in China [2], [14].

There are two potential limitations regarding the previous assessments of the success of the WBLP strategy. One is that previous studies only evaluated the short-term impact of the WBLP strategy [9], [10]. The WBLP's control strategy focused on the large-scale use of chemotherapy to control morbidity in humans and livestock, an approach which has been frequently questioned regarding its sustainability. For example, the compliance rate of chemotherapy can decrease to a great extent because of fatigue with repeated treatments [6], [15]. The long-term impact of the WBLP strategy could be different from the short-term situation. The second potential limitation is that earlier assessments were only based on a non-spatial perspective. That is, they used the magnitude of the absolute number of cases to evaluate the overall control effect, but neglected to consider the spatial aspects which can provide some new and even different results. It is well known that a certain number of cases in a region could be due to two completely different scenarios, a clustered risk profile with cases concentrated in only a few areas and a random risk profile with cases occurring nearly randomly throughout the region. Different risk patterns require a distinct control strategy and decision-making process. So evaluating the long-term impact of the WBLP strategy from a spatial perspective would be valuable for future planning of schistosomiasis control.

In this study we used the national datasets from the five provinces in the lake and marshland regions from two periods, 1999–2001 and 2007–2008, with the aim of assessing the long-term impact of the WBLP strategy on schistsomiasis. We compared the changes of the spatial risk distribution of schistosomiasis between the two study periods and contrasted the compositions of the two random effects of spatially correlated and spatially independent heterogeneities.

## Materials and Methods

### 2.1 Study area

Our study was carried out in the lake and marshland regions of schistosomiasis in the middle and lower reaches of the Yangtze River, which included the five provinces of Hunan, Hubei, Anhui, Jiangxi and Jiangsu. According to the latest report (2009) on the schistosomiasis situation, it was estimated that 98.7% of the snail-infested areas and 97.8% of the *S. japonicum* infected people in China were concentrated in these five provinces [16]. These provinces included 261 schistosomiasis endemic counties. Of these, 115 reached the criterion of *transmission interruption*, 57 achieved *transmission control* and 89 had *ongoing transmission* [16], [17]. It is obvious that the lake and marshland regions should be the focus for China's schistosomiasis control program.

The WBLP project started in 1992. It was completed at the end of 1998 in Anhui, Jiangxi, and Jiangsu provinces and continued until the end of 2001 in Hubei and Hunan. The three provinces that completed the project in 1998 continued to carry out schistosomiasis control activities using their own funds and according to the operational plan set out by the WBLP [10]. Thus, these provinces underwent similar stages of the schistosomiasis control strategy.

### 2.2 Parasitological data

The county-level prevalence data on *S. japonicum* infection in the lake and marshland regions were obtained from the national annual report on schistosomiasis. These data were first collected through village-based field surveys using a two-pronged diagnostic approach (screening by a serological test on all residents of 5 to 65 years old and then confirmation by a parasitological test), then reported to the towns and finally summed at the county level, with only the county-level totalized databases made available to us. This system of recording and annual reporting had been in place since 1999 [18]. The diagnostic criteria and diagnostic approaches for schistosomiasis cases were as per the national guidelines [19].

Prior to and including during 2004, these data were maintained and managed by Fudan University (formerly Shanghai Medical University). Beginning 2005, this task was taken over by the National Institute of Parasitic Diseases in Shanghai (Formerly the Institute of Parasitic Diseases), Chinese Center for Disease Control and Prevention. The national databases for the lake and marshland regions during the two periods of 1999–2001 and 2007–2008 were obtained from the corresponding institutes, respectively. The total number of schistosomiasis cases and population at risk in each county were used to estimate the prevalence of schistosomiasis and to analyze the dynamics of spatial risk distribution and spatial heterogeneities of schistosomiasis-related risk between the two study periods.

### 2.3 Administrative area and hydrological base maps

County-based digitized polygon maps in the lake and marshland regions were obtained for the five study provinces [5], [6]. Digitized maps of the Yangtze River and Dongting and Poyang Lakes were also obtained. Attribute data on schistosomiasis were linked to the county maps to establish the spatial database and facilitate the visualization of the results. During the 10-year study period, the administrative boundaries of counties changed slightly. To simplify the analysis and for comparability of the results, the administrative divisions in 2008 were used as the standard and data from the other study years were modified accordingly. Two types of topological manipulations were involved, the merging and splitting of polygons, which have only ignorable impacts on this study because the county-level data is much too unspecific to really capture the dynamics of schistosomiasis. The former operation combined two or more polygons into a single, new polygon; and the number of schistosomiasis cases and the population at-risk were then summed to produce the new polygon's disease data. The latter operation divided one polygon into two or more new polygons, in which the number of schistosomiasis cases in each new county was estimated using the proportion of the population of the original county that was present in each of the new counties. All data manipulation was undertaken within ArcGIS9.2 software (Environmental Systems Research Institute, Inc., Redlands, CA, USA).

### 2.4 Statistical analysis

The analysis consisted of four procedures. Firstly, crude prevalence of schistosomiasis was calculated and summarized for those counties with reported cases using conventional descriptive statistics (e.g., median and quartiles).

Secondly, the counties in the lake and marshland regions were classified into five classes according to the dynamics of their prevalence status: 1.) non-endemic counties where no cases were reported during the two study periods; 2.) unchanged endemic counties where schistosomiasis cases were continuously reported across years; 3.) disappeared endemic counties where cases were continuously reported in 1999–2001, but no cases were reported in 2007–2008; 4.) newly appeared endemic counties where cases were continuously reported in 2007–2008, but no cases were reported in 1999–2001; and 5.) fluctuating endemic counties where cases were reported in one or two years of 1999–2001 and one year of 2007–2008. Maps of these categories of endemicity were created using ArcGIS9.2 software.

Thirdly, a Bayesian random-effect model-which was first introduced by Clayton and Kaldor in 1987 [20] and developed further by Besag et al. in 1991 [21]- was built to analyze the spatial distribution of schistosomiasis for different time points. In this model for estimating relative risk (RR), area-specific random effects are decomposed into two latent components: one component represents the effects of schistosomiasis-related risk factors that vary in a structured manner in space (referred to as correlated heterogeneity, CH) and another component indicates the effects from schistosomiasis-related risk factors that vary in an unstructured way among areas (referred to as uncorrelated heterogeneity, UH). The model is formulated as [22],
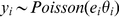
(1)

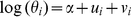
(2)where 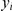 is the number of reported schistosomiasis cases in county *i*; 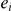 is the expected number of schistosomiasis cases in county *i*; 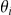 is the expected or predicted RR in county *i*; 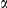 is the overall level of risk assuming the effects of UH and CH are zero; 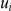 is the correlated heterogeneity (CH) which was modeled using the conditional autoregressive (CAR) structure and 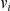 is the uncorrelated heterogeneity (UH) that was modeled using a Gaussian distribution, for which the following formulas are used, respectively:
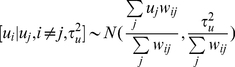
(3)

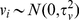
(4)where 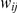 is the weight of the neighbor *j* for area *i*, 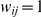 if *i*, *j* are neighbors, otherwise 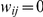; 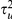 and 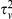 are precisions and their inverses are the variance of 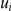 and 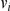, respectively.

Bayesian methods were used to fit the spatial model, implemented using WinBUGS1.4.1 software (Imperial College and MRC, London, UK). Parameters 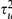 and 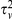 control the variability of CH and UH effects, for which prior distributions were specified using the same vague gamma prior distributions: Gamma(0.5,0.0005). For the baseline risk 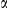, a vague normal prior distribution: *N*(0,0.0001) was used. Model fitting was carried out using two separate chains starting from different initial values, and 30,000 iterations were run: the first 10,000 samples were discarded as burn-in and the remaining 20,000 iterations from each chain were used for parameter estimation. Convergence was checked by visual examination of the time series plots of samples from each chain and by computing the Gelman and Rubin diagnostic statistic.

Finally, the estimated parameters of the model (overall risk 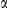, the variation of the UH and CH components) were summarized in a table. The predicted RR, the posterior probability of RR>1, the ratio of UH to CH and the model residuals were exported and linked with the digitized polygon maps using ArcGIS9.2 software to display their spatial distributions.

## Results

The number of counties reporting schistosomiasis cases and the overall crude prevalence increased slightly from 1999 to 2001 during the later period of the WBLP strategy and then decreased during 2007–2008, but the prevalence was still higher than that in 1999. The overall variation (95% CI) in the prevalence showed a tendency of continuous decline except for a slight rebound in 2001 (Table 1).

**Table 1:** Crude prevalence of schistosomiasis japonicum in the lake and marshland regions, China*.

Year	N	Min (10^−6^)	Median (10^−3^)	*P*2.5 (10^−5^)	*P*97.5 (10^−2^)	Max (10^−2^)
1999	144	9.83	3.76	2.32	7.52	25.0
2000	147	14.8	5.25	11.4	6.03	11.0
2001	160	42.2	7.50	16.1	6.76	21.3
2007	143	4.90	4.12	3.05	4.35	8.54
2008	137	4.90	4.12	1.83	4.00	8.65

*N: number of counties reporting schistosomiasis cases;

*P*2.5: percentile 2.5; *P*97.5: percentile 97.5.

**Figure 1:**
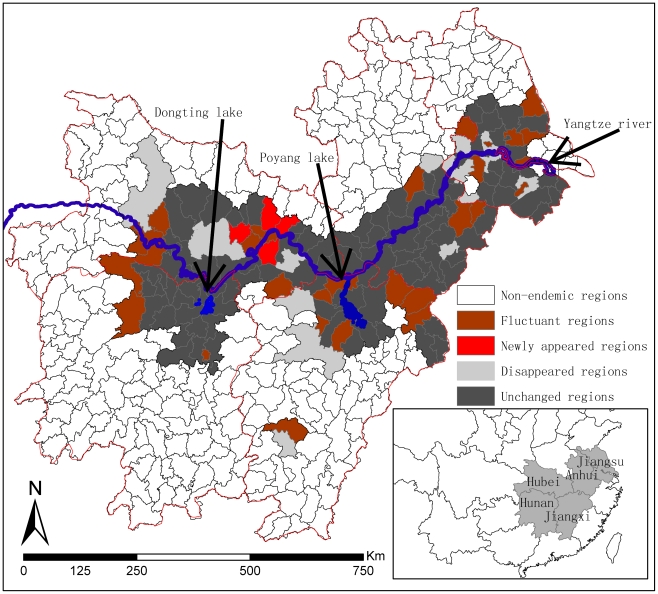
Spatial distribution of different types of schistosomiasis endemic counties. It shows the distribution of the unchanged, disappeared, new appeared and fluctuating endemic counties in different colors. The red lines are the boundaries of the studied five provinces.

Figure 1 shows the distribution of the unchanged, disappeared, new appeared and fluctuating endemic counties. The number (proportion) of endemic counties for each type were 110 (64.71%), 25 (14.71%), 4 (2.35%), and 31 (18.24%), respectively. The four newly appeared endemic counties in 2007–2008 were all located in Hubei province. Different types of endemic counties were intermingled along the Yangtze River and the Poyang and Dongting Lakes. Exceptions are one fluctuating and one disappeared endemic county, which were isolated and located in Jiangxi province. Besides, the fluctuating and disappearing counties are mainly on the geographical margins of the endemic areas.

From Table 2, we see that the overall risk of schistosomiasis in the counties tended to decrease gradually and that the risk in 2008 was reduced to less than half of the risk in 1999. The changes in variation of the CH and UH components were different. The former fluctuated across years and reached a peak in 2008 whereas the latter first decreased from 1999 to 2001, then increased again from 2007 and finally rebounded to a level that was similar to 1999. Except for the year of 1999, the variation in the CH component was greater than the variation in the UH component.

**Table 2:** Estimates of overall risk of schistosomiasis japonicum and the magnitude of correlated and uncorrelated heterogeneities*.

Year	Mean	Std (CH)	Std(UH)
1999	0.34(0.17,0.55)	1.67(0.02,7.49)	3.33(2.48,4.10)
2000	0.28(0.11,0.55)	3.65(0.03,6.56)	2.75(1.60,4.05)
2001	0.30(0.15,0.57)	3.64(2.58,4.80)	1.77(0.96,2.87)
2007	0.18(0.11,0.35)	2.98(0.02,6.90)	2.97(2.04,4.13)
2008	0.14(0.04,0.43)	5.91(1.06,7.79)	3.22(2.31,4.79)

*The values in the parentheses are the 95% CI of the estimates.

**Figure 2:**
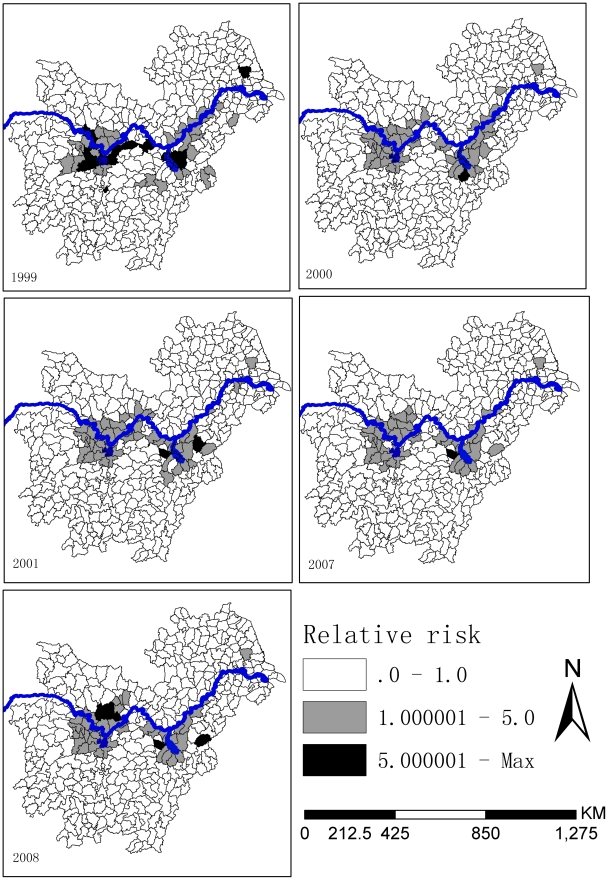
Posterior relative risks for schistosomiasis. The risk degrees are distinguished on the basis of gray levels. The black shading denotes the highest degree of risk, while white is the least risk.

The magnitude of the relative risk generally decreased during the study period and the counties with posterior expected RR>1 were mainly located in the areas surrounding Poyang and Dongting Lakes. In the southern part of Yangtze River, the areas with RR>1 were relatively stable in space; while in the northern part of Yangtze River, the spatial distribution of predicted risk counties spread northwards around the Dongting Lake, but was compressed in areas near Poyang Lake (Figure 2).

**Figure 3:**
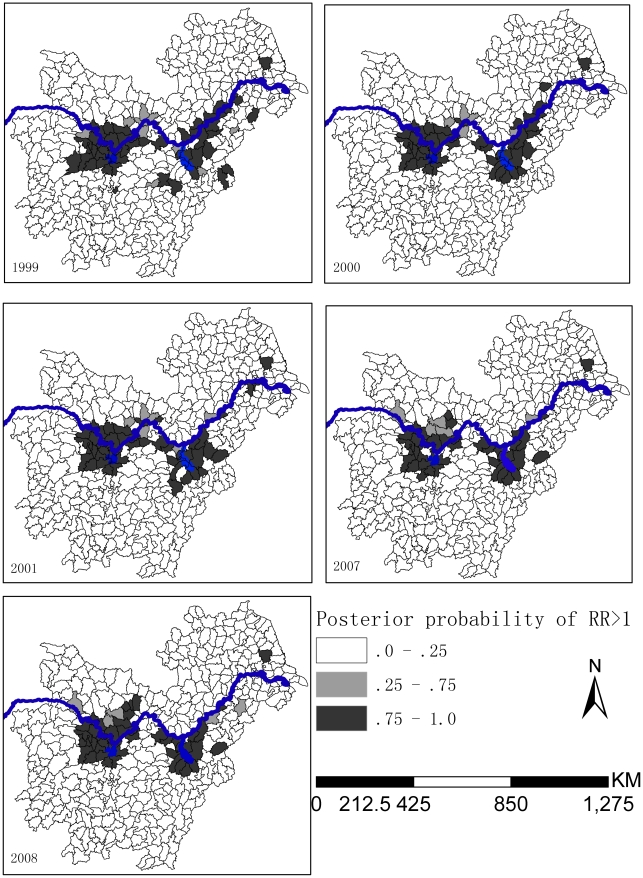
Posterior probability of RR>1. The posterior probabilities are distinguished on the basis of gray levels. The black shading denotes the highest probability, while white is the least probability.

Figure 3 shows that the counties with the highest probability of greater than average risk (i.e., RR>1) were mostly confined to Poyang and Dongting Lakes and their neighboring counties. For the counties where the posterior probability was high in the lower reaches of the Yangtze River in 1999–2001, the posterior probability was reduced in 2007–2008.

**Figure 4:**
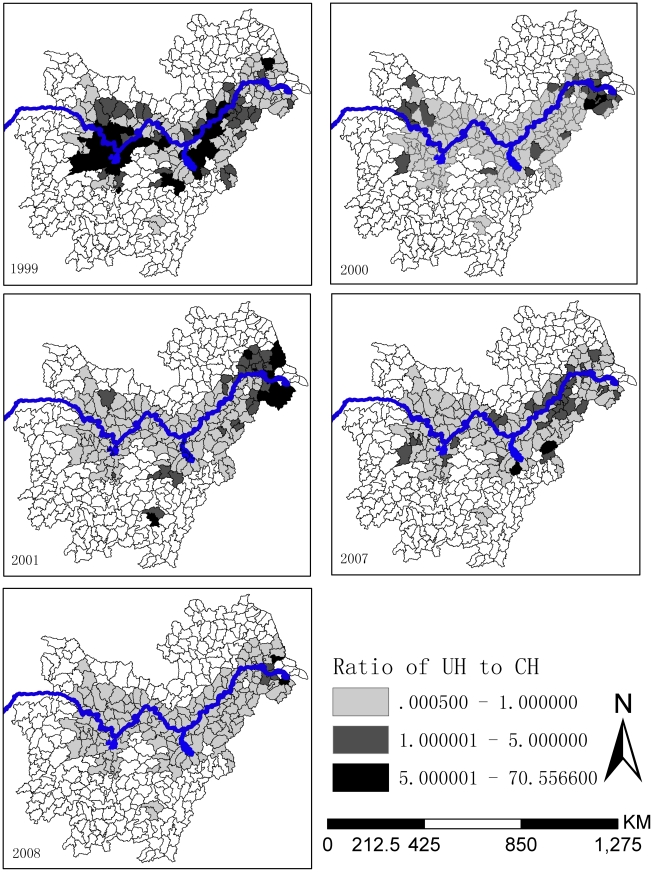
Ratios of the random effects of uncorrelated heterogeneity (UH) to correlated heterogeneity (CH). The ratios of the random effects of UH to CH are distinguished on the basis of gray levels. The black shading denotes the highest ratio, while white is the least ratio.

In the endemic regions around the Poyang and Dongting Lakes, schistosomiasis risk was dominated by the UH effects of schistosomiasis-related risk factors in 1999, but the CH effects were dominant in the other years. In contrast, in the lower reaches of Yangtze River the primary component of the heterogeneity effects for schistosomiasis risk was relatively unstable (Figure 4).

The model residuals ranged between −1.40 and 1.65. No obvious outliers were identified, so the distribution of the residuals after adjusting for the effects of the UH and CH components were not displayed here (data are available upon request).

## Discussion

This study presents an application of Bayesian methods to evaluate the long-term impact of the chemotherapy-based WBLP strategy on the spatial distribution of schistosomiasis japonicum. It is widely reported that the transmission dynamics of schistosomiasis are closely related to socio-economic, climatic, demographic, biological and environmental factors [23], [24], [25]. When studying the epidemiology of schistosomiasis and evaluating the impact of control strategies, it is impossible to consider all the potential risk factors related with interested diseases because either the information is unavailable or disease mechanisms are unclear. Hence, previous reports have only included the most important factors or those of specific interest [4], [5], [26], [27], [28], [29], [30], [31], which will contribute to bias in effect estimates because of unadjusted effects from risk factors that have been ignored. From the spatial perspective, all the potential risk factors related with studied diseases can be divided into two latent components of random effects, spatially correlated heterogeneity (CH) and uncorrelated heterogeneity (UH). This idea has been mathematically demonstrated within the well known Besag, York and Mollié model [21], which permits us not only to analyze and predict the disease risk more accurately, but also to study and compare the dynamics of the two latent components. For schistosomiasis, CH could encompass the combined effects of all the unmeasured environmental factors (e.g., normalized difference vegetation index (NDVI), land surface temperature (LST), rainfall), socio-economic factors (e.g., income), and the biological factors of the intermediate host *Oncomelania hupensis* (e.g., density of snails), which are affected by the other two factors. Among others, the snails are the focus for schistosomiasis control, so the transmission control strategies are closely related with CH such as mollusciciding, environmental modification and liming the banks along canals and drainage ditches. Unfortunately, this was not the focus for WBLP's schistosomiasis control strategy. By contrast, UH could encompass spatially unstructured risk factors, which might include demographic factors (e.g., age, gender, education level, and occupation), human behavioral factors (e.g., frequency of contact with infected water, personal protections) and livestock-related factors. Here, the reservoir hosts (human and cattle) are the intervention targets and the morbidity control strategy with chemotherapy-based population treatment emphasizes the observed UH. Hence, the dynamic changes in CH and UH can help signify the control effect of schistosomiasis to a certain degree and direct future control emphasis.

From our study, we see that the prevalence of schistosomiasis has been greatly reduced and maintained at a low level. The prevalence during 2007–2008 was reduced further, but still in the same order of magnitude (10^−3^). This suggests that the chemotherapy-based WBLP strategy has had little effect under the low endemic levels of schistosomiasis in China and some new control strategies are needed [32], [33]. Also, we found that the spatial distribution of schistosomiasis risk during 2007–2008 was only slightly reduced and <10% endemic counties in 1999–2001 were declared to be free of cases in 2007–2008. The most severely affected areas were located along the Yangtze River, in the areas of the great lakes (Dongting and Poyang Lakes), and their surroundings [1], [34], [35], where the predicted RR>1 and its posterior probability were high. The spatial distribution of current schistosomiasis risk seemed to be stable and was not obviously compressed in space. This was confirmed by the intersecting distributions of fluctuating, newly appeared, disappeared and unchanged endemic counties. Combined with the above results, we may conclude that continuing a chemotherapy-based control strategy following the decade-long WBLP is likely to contribute little to further schistosomiais control under the current situation of low morbidity levels. We also conclude that it is difficult to further restrict the spatial distribution of schistosomiasis endemic regions using current control methods. The rebounding of prevalence during 1999–2001 and the increasing RR in some counties during 2007–2008, which were partly due to reduced compliance with drug use and the persistence of extensive snail habitats, suggests that the impact of the WBLP strategy was inconsistent. This has been frequently highlighted by many researchers who suggest that snail control should be given more attention for sustainable control of schistosomiasis [2], [5], [6], [14], [36].

The variation in UH was decreased from 1999 to 2001 and then increased again from 2007 to 2008, reflecting a good short-term control effect of chemotherapy-based WBLP strategy, but the long-term effect may not be optimal. The variation in CH showed a tendency of increasing and in 2008 it was over 3 times as that of 1999. This may suggest the risk from the intermediate host snails increased continuously, possibly because snail control was not emphasized within the WBLP strategy. For the predicted high risk regions around the Dongting and Poyang Lakes, the ratios of UH to CH were over 1 only occurred in 1999, suggesting that CH has become the main component of current schistosomiasis risk in those regions where transmission control strategy focusing on snail control should be implemented. While for the lower reaches of Yangtze River, it is more complicated for the changes of UH to CH. The possible reason is the control effect was not only affected by itself, but also was influenced by the schistosomiasis epidemics in the upriver provinces where the cercaria could be brought to the downriver provinces following the water flows. So morbidity control strategy in the lower reaches of Yangtze River should be maintained until the snails in the upriver provinces have been well controlled.

Besides, the frequent flooding of the Yangtze River, water resource development projects (e.g., Three Gorges Dam Project and South-to-North water transfer project) [37], [38], climate change/global warming, anti-flood policies (returning reclaimed land to lake, leveling dykes between main levees and building new towns for resettlement), mobility of populations, the frequent trade of livestock and increased tourism and travel to endemic regions are all important drivers for the fluctuation, (re)-emergence and spread of schistosomiasis and contribute to the continuing challenge of schistosomiasis control, especially sustainable control. Experiences in China and Japan indicate that controlling the intermediate host snail should result in a more sustainable impact compared to other control approaches [17], [39]. The primary CH component of current schistosomiasis risk identified in this study also implies that a future control strategy should shift to transmission control strategy with snail control as an emphasis, which would have a sustainable impact on controlling schistosomiasis and be helpful for facilitating the ultimate elimination of schistosomiasis in China.

There are two potential shortcomings in our study that warrant discussion. One limitation is that the quality of reported schistosomiasis data from different regions may be inconsistent. Case data were collected through a bottom-up disease reporting system aimed at monitoring the national disease status, so the data reliability was largely based on the quality of data reported by local institutions. Another limitation is that the diagnostic approach for schistosomiasis is not 100% sensitive and specific. The parasitological test (e.g., Kato-Katz technique) has a low sensitivity, while serological tests (e.g., indirect hemagglutination assay, IHA) have low specificity [40]. According to expert opinion, the estimated sensitivity and specificity of the Kato-Katz technique are about 20–70% and 95–100%, respectively and for the IHA technique about 90–95% and 85–90%, respectively [41]. Therefore, some correction methods for the prevalence estimates are needed for a precise analysis. Wang et al. (2008) reported a simple Bayesian approach to correct the reported prevalence of schistosomiasis by considering the uncertainties of the diagnostic approaches [42], but they assumed that the diagnostic methods had the same uncertainties for all levels of endemicity. In fact this is not the case and it could introduce other biases to the results. The best possible solution may be to develop a correction method weighted according to prevalence, where the determination of appropriate weights is the most important issue. This is an area of future work.

In conclusion, we conducted a comparative study of the spatial epidemiology of schistosomiasis using two datasets from 1999–2001 and 2007–2008 in the lake and marshland regions to evaluate the long-term impact of WBLP strategy on China's schistosomiasis status. The WBLP strategy appears to have had a good short-term impact on schistosomiasis control, but its long-term effect has not been ideal. It has successfully reduced the morbidity of schistosomiasis to a low level, but can not contribute further to current schistosomiasis control considering the low endemic level in China. The WBLP strategy has also failed to reduce the geographical range of affected counties. To achieve this and for sustainable control, transmission control strategies focusing on snails should become the next priority in the two most seriously affected regions surrounding the Dongting and Poyang Lakes. Whereas in the lower reaches of the Yangtze River, WBLP's morbidity control strategy should be continued for some time until the snails in the upriver provinces are well controlled.
